# Machine-learning-based global optimization of microwave passives with variable-fidelity EM models and response features

**DOI:** 10.1038/s41598-024-56823-7

**Published:** 2024-03-15

**Authors:** Slawomir Koziel, Anna Pietrenko-Dabrowska

**Affiliations:** 1 Engineering Optimization & Modeling Center, Reykjavik University, 102 Reykjavík, Iceland; 2 Faculty of Electronics, Telecommunications, and Informatics, Gdansk University of Technology, 80-233, Gdańsk, Poland

**Keywords:** High-frequency engineering, Globalized optimization, Surrogate-based design, Variable-fidelity models, Nature-inspired algorithms, EM-driven design, Response features, Engineering, Electrical and electronic engineering

## Abstract

Maximizing microwave passive component performance demands precise parameter tuning, particularly as modern circuits grow increasingly intricate. Yet, achieving this often requires a comprehensive approach due to their complex geometries and miniaturized structures. However, the computational burden of optimizing these components via full-wave electromagnetic (EM) simulations is substantial. EM analysis remains crucial for circuit reliability, but the expense of conducting rudimentary EM-driven global optimization by means of popular bio-inspired algorithms is impractical. Similarly, nonlinear system characteristics pose challenges for surrogate-assisted methods. This paper introduces an innovative technique leveraging variable-fidelity EM simulations and response feature technology within a kriging-based machine-learning framework for cost-effective global parameter tuning of microwave passives. The efficiency of this approach stems from performing most operations at the low-fidelity simulation level and regularizing the objective function landscape through the response feature method. The primary prediction tool is a co-kriging surrogate, while a particle swarm optimizer, guided by predicted objective function improvements, handles the search process. Rigorous validation demonstrates the proposed framework's competitive efficacy in design quality and computational cost, typically requiring only sixty high-fidelity EM analyses, juxtaposed with various state-of-the-art benchmark methods. These benchmarks encompass nature-inspired algorithms, gradient search, and machine learning techniques directly interacting with the circuit's frequency characteristics.

## Introduction

Over the years, the performance requirements formulated for high-frequency systems, including microwave passive components, have increased significantly. Fields like mobile communications^1^, radio-frequency identification^2^, implantable systems^3^, internet of things^4^, or energy harvesting^5^, require structures that can be reconfigured^6^, enable broadband^7^ or multi-band operation^8^, harmonic suppression^9^, but also feature small physical dimensions^10–12^. The circuits delivering the aforementioned and other functionalities exhibit complex topologies, typically parameterized by large numbers of variables, mainly geometrical ones. Their layouts are often densely arranged, especially in the case of miniaturized components, obtained using approaches like transmission line meandering^13^, slow-wave phenomena^14^, or integrating extra components (e.g., resonators^15^, stubs^16^, etc.). Precisely characterizing geometrically intricate microwave circuits necessitates full-wave electromagnetic (EM) analysis. Unlike conventional models (analytical^17^, equivalent networks^18^), EM simulations can accurately capture phenomena like cross-coupling, dielectric and radiation losses, and the effects of environmental components (e.g., SMA connectors). As a result, the use of electromagnetic tools has become indispensable today. They are extensively utilized across all design phases, from shaping the geometry^19^, conducting parametric studies^20^, to finalizing the design^21^.

One of the most prevalent simulation-driven processes involves adjusting geometry parameters, yet it is a costly endeavour. Numerical optimization algorithms demand numerous system evaluations, making it an expensive task. Even with local optimization methods such as gradient-^22^ or stencil-based search^23^, the expenses can quickly accumulate, often requiring many dozens or even hundreds of EM analyses. Interactive methods (experience-driven parametric studies^24^) are cheaper, yet unable to handle more than one or two parameters at a time, let alone several design goals and constraints. On the other hand, for a growing number of cases, it is at least recommended if not necessary, to employ global search methods. Examples include optimization of frequency selective surface^25^, coding metasurfaces^26^, metamaterial-based components^27^, antenna pattern synthesis^28,29^, design of conformal^30^ or sparse arrays^31^, as well as multi-objective design^32^. Other instances include, among various others, redesigning microwave components across extensive ranges of operating conditions^33^, and optimization of compact circuits^34^. For the latter, redundancy of geometry parameters introduced by using various miniaturization strategies (line folding^35^, CMRCs^36^) makes the relations between the circuit dimensions and its electrical characteristics rather unintuitive.

Currently, population-based algorithms inspired by nature^37–40^ have taken the lead in global optimization. They are implemented to mimic various biological^41^ or social^42^ phenomena, including biological evolution^43^, feeding or hunting strategies of various species^44,45^, etc. The fundamental mechanisms involve information exchange among the individuals (particles, agents, members)^46^ within the pool of potential solutions (swarm, population, pack)^47^ for the specific problem. This exchange can impact the individuals by altering their composition (through crossover, mutation^48^), or by repositioning them within the search space. For instance, this might involve introducing a randomized bias towards the best solution discovered thus far, whether individual or overall^49^. Practical observations confirm global search capability achieved using such means although there is little of no supporting theory. Further, the computational efficiency of population-based algorithms is poor. A typical algorithm run requires thousands of objective function evaluations, which essentially rules out direct nature-inspired optimization of EM simulation models unless the evaluation time is short (e.g., up to 10–30 s per analysis). Common techniques within this category comprise genetic^50^ and evolutionary algorithms^51^, particle swarm optimization (PSO)^52^, differential evolution^53^, firefly algorithms^54^, ant systems^55^, grey wolf optimization^56^. Although every year witnesses new development^57–60^, it seems that—despite fancy names—the new algorithms offer incremental adjustments of the existing techniques.

Enabling nature-inspired EM-driven optimization requires acceleration, which can be achieved using surrogate modelling methods^61,62^. Several widely used techniques in this context involve Gaussian process regression (GPR)^63^, kriging^64^, neural networks^65^, and polynomial chaos expansion^66^. Typically, the surrogate model serves as a predictor, determining optimal design locations, and continuously updates using full-wave simulation results gathered in the course of the search process^67^. The criteria for infilling depend on the objective, such as enhancing model accuracy (maximizing mean squared error reduction^68^), identifying the global optimum (minimizing predicted objective function^69^), or balancing exploration and exploitation^70^. The search procedures of this class are often referred to as machine learning methods^71–73^. The fundamental issue with surrogate-assisted methods is building the metamodel. Modelling the behaviour of microwave circuit responses, usually represented as frequency characteristics, becomes challenging due to their highly nonlinear nature. Covering extensive frequency ranges and accounting for diverse geometry/material parameters demands substantial training datasets. Many of machine learning frameworks in the realm of high-frequency design are therefore demonstrated for systems defined over small parameters spaces, either in terms of dimensionality or parameter ranges^74,75^. These issues can be mitigated using variable-fidelity simulations^76^. Other potential acceleration techniques include constrained modelling^77–79^, and the response feature technology^80^; however, no machine learning approach incorporating these methods has been described in the literature thus far. Yet another approach that might be useful for enhancing dependability of data-driven modelling is data normalization, which is normally applied to transform the features of the system at hand to be on a similar scale. This contributes to the improvement of the performance and model training stability. Some of popular normalization and data augmentation techniques include min–max normalization, Z-score normalization^81^, decimal scaling, or class-balancing (e.g., synthetic minority oversampling technique, SMOTE^82^, oriented towards oversampling minority classes thereby improving the balance of the dataset).

This study introduces a machine-learning framework aimed at accelerating the global optimization of microwave passive components, also miniaturized structures. The approach tackles the design challenge by focusing on response features—a discrete set of points derived from EM-simulated outputs that quantify the circuit's performance specifications. Leveraging the weakly-nonlinear dependence of feature point coordinates on geometry/material parameters enables efficient surrogate modelling. The algorithm's initial phases, i.e., parameter space pre-screening and surrogate model construction, utilize low-fidelity EM models. The core iteration involves generating infill points and refining the surrogate model, primarily based on the co-kriging approach. This method combines low-fidelity data with high-fidelity EM analyses carried out at this stage. By employing characteristic points and multi-resolution EM analysis, the computational efficiency is noteworthy: the average optimization cost is around sixty high-fidelity EM simulations for the circuit under design. Concurrently, the quality and consistency of designs compete favourably against benchmarks, which encompass multiple-start gradient search, nature-inspired optimization, and kriging-based machine learning directly working with circuit frequency responses. This highlights the significance of response features in enhancing both reliability and computational efficiency within the proposed algorithm.

## Global machine-learning microwave optimization using response features and multi-resolution computational models

This section delves into the intricacies of the proposed optimization approach. "Microwave design optimization. Problem statement", “Response feature methodology”, "Variable-fidelity computational models" sections revisit the formulation of the design task, the concept of characteristic points, and variable-fidelity simulation models, respectively. A concise overview of kriging and co-kriging surrogates is presented in "Surrogate modelling using kriging and co-kriging" section. The optimization process begins with the pre-screening stage, detailed alongside the construction of the initial (kriging) surrogate model in "Parameter space pre-screening. Initial surrogate model construction" section. "Generating infill by means of nature-inspired optimization. Co-kriging surrogate" section expounds on the machine-learning framework involving co-kriging models and the infill criterion based on predicted objective function improvement. Lastly, "Complete optimization framework" section encapsulates the entire procedure using the pseudocode and a flow diagram.

### Microwave design optimization. Problem statement

**Figure 1:**
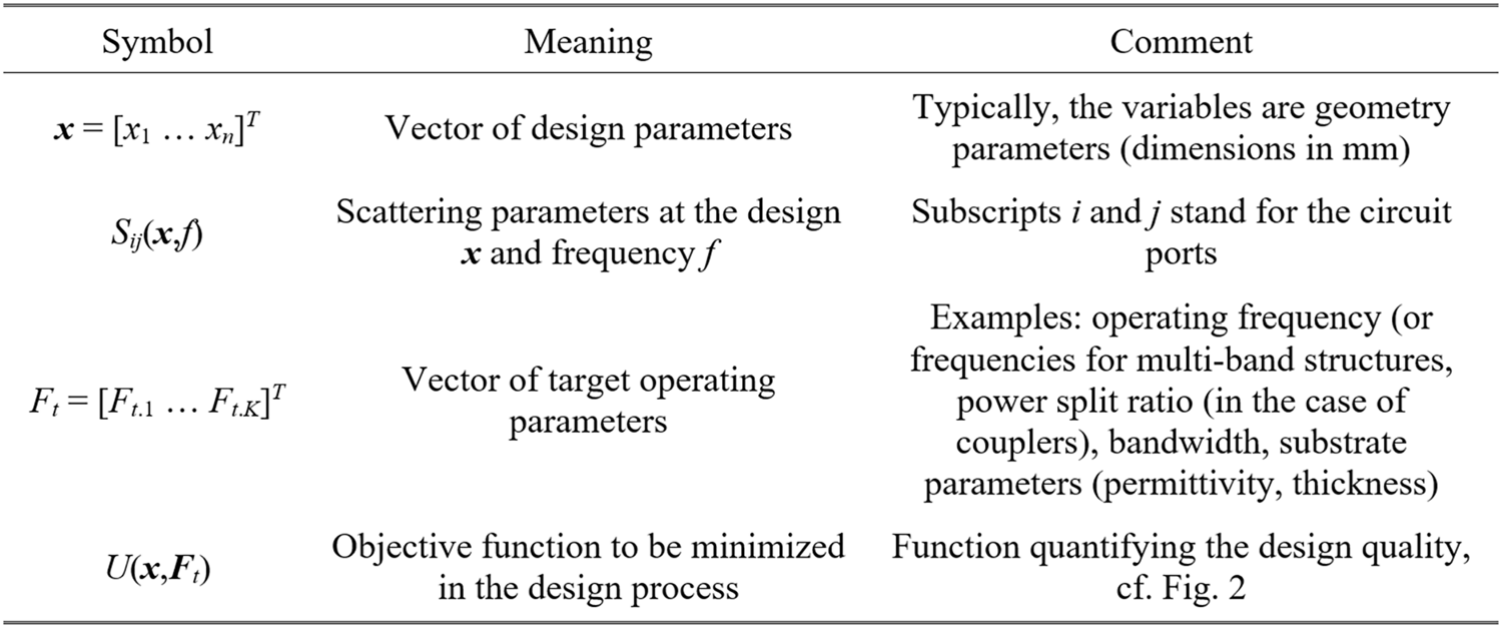
Microwave design optimization: notation and terminology.

**Figure 2:**
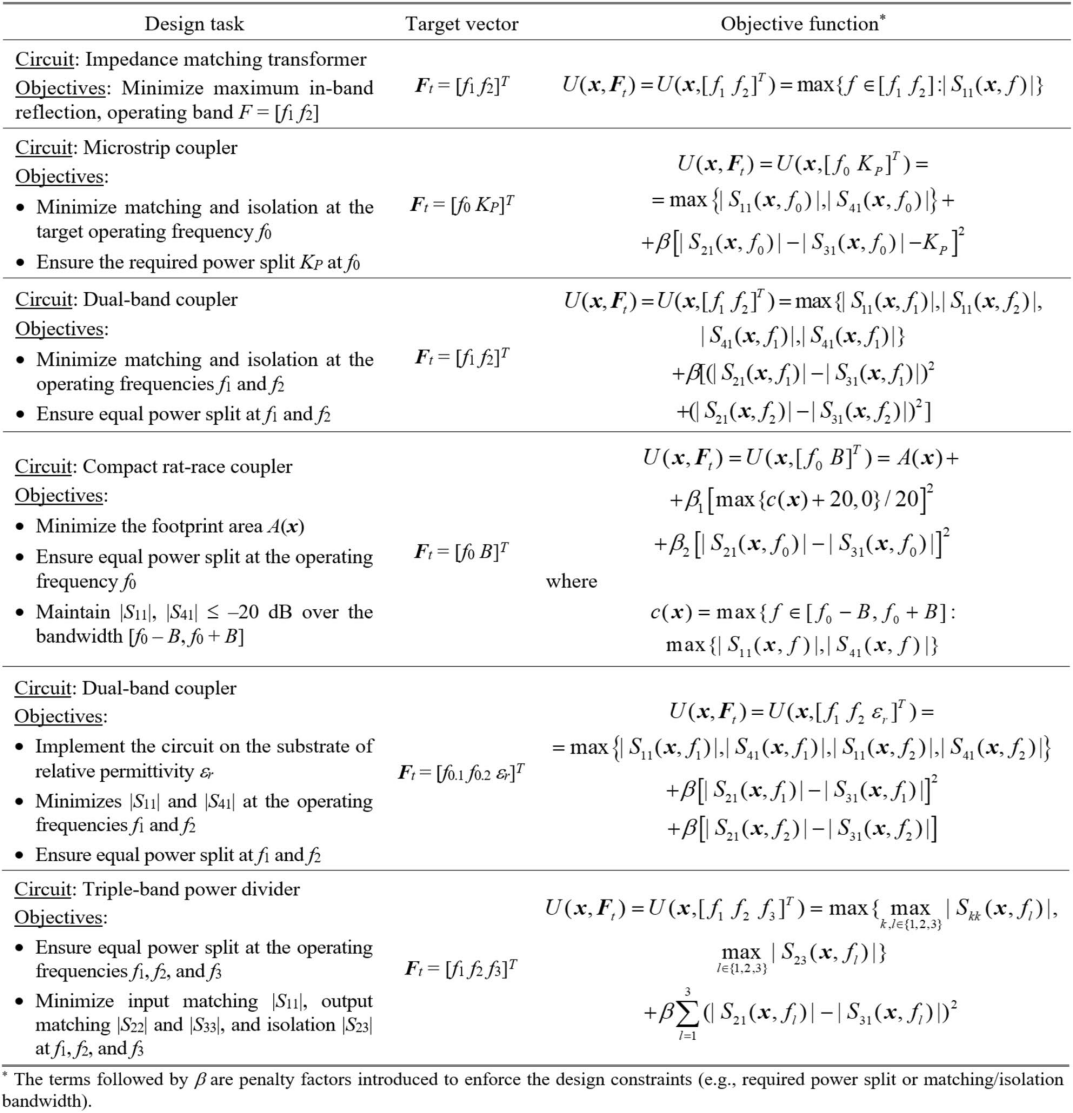
Examples of microwave design optimization problems. Verbal description of the task (left) is followed by a definition of the target vector ***F***_*t*_ (middle), and a possible definition of the objective function (right).

In this study, optimizing microwave circuits involves adjusting their independent variables, typically geometry parameters like component widths, lengths, and their spacings, consolidated within the parameter vector ***x*** (as illustrated in Fig. 1). The objective vector ***F***_*t*_ comprises the design goals, encompassing target operating parameters such as center frequency, power split ratio, and bandwidth. Evaluating the design ***x*** in relation to the target vector ***F***_*t*_ is accomplished through a scalar objective function *U*(***x***,***F***_*t*_). Figure 2 provides several examples of common design scenarios along with the corresponding definitions of *U*. The primary type of microwave circuit responses are scattering parameters (cf. Fig. 1)^83^, along with the quantities that can be derived therefrom (e.g., the phase characteristic).

It is important to highlight that most real-world design challenges involve multiple objectives; that is, there is a need to enhance or regulate more than just one parameter or quantity. As most of available optimization algorithms are single-objective ones, multi-criterial problems are normally reformulated, e.g., by casting all but the primary objective into constraints^84^. Another popular option is scalarization using, e.g., a linear combination of goals^85^. Genuine multi-objective design is outside the scope of this paper.

Now, we can express the microwave optimization task as a minimization task represented by the following form:

1 $$ {\varvec{x}}^{*} = \arg \mathop {\min }\limits_{{{\varvec{x}} \in X}} U({\varvec{x}},{\varvec{F}}_{t} ) $$

where x^*^ represents the sought-after optimal design, and *X* denotes the parameter space, typically an interval determined by the lower and upper variable bounds for *x*_*k*_, *k* = 1, …, *n*.

### Response feature methodology

**Figure 3:**
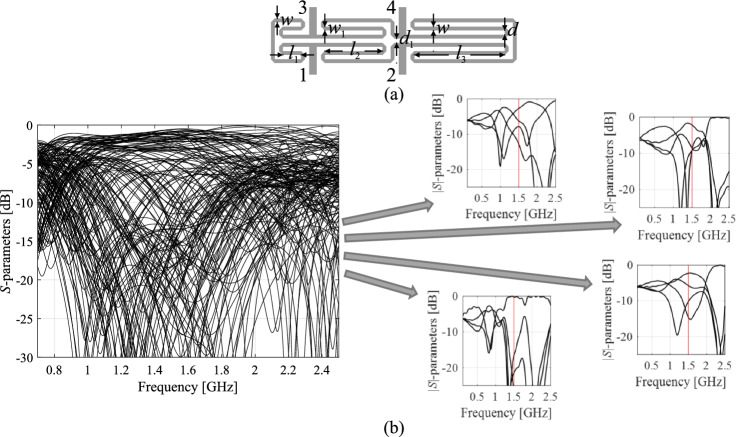
Miniaturized rat-race coupler and its scattering parameters: (**a**) circuit architecture, (**b**) scattering parameters at randomly selected designs. If local search based on the objective function (as illustrated in Fig. 2, with the target frequency set at 1.5 GHz and marked using vertical lines) were initiated from many of the shown designs, it would prove unsuccessful due to significant misalignment between the target and the actual operating conditions.

Full-wave computational models ensure reliable evaluation of microwave components, but they are CPU-intensive. This is a major hindrance to optimization procedures. The nonlinearity of circuit responses poses additional obstacles to globally exploring the parameter space. Figure 3 shows examples of frequency characteristics of a microstrip coupler at a number of randomly generated designs. Circuit optimization for a centre frequency of 1.5 GHz requires global search as local tuning initiated from most of the shown designs would not succeed.

The response feature techniques^86^ were introduced to address the mentioned challenges by redefining the design problem using a discrete set of characteristic points (or features) from the system outputs^87^. This method leverages straightforward connections between the circuit's geometry/material parameters and the frequency and level coordinates of these points^80,86–89^. Consequently, it regularizes the objective function, hastening optimization processes^87^, and simplifying behavioural modelling^89^.

**Figure 4:**
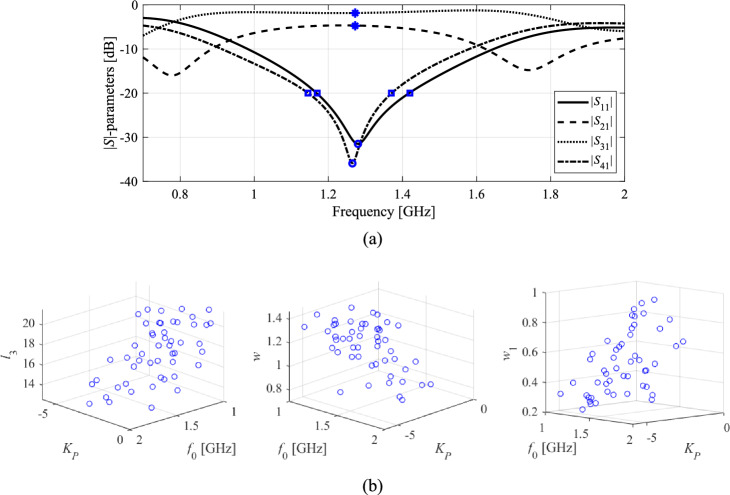
Characteristic points selection for a microwave coupler: (**a**) possible feature point choices: open circle—points corresponding to the minima of the matching and isolation characteristics, *—points corresponding to the power split ratio (evaluated at the frequency *f*_0_ being the average of the frequencies of |*S*_11_| and |*S*_41_| minima), open square—points corresponding to − 20 dB levels of |*S*_11_| and |*S*_41_|; (**b**) relationship between operating conditions (extracted from the response features, here the center frequency *f*_0_ and power split ratio *K*_*P*_) and selected geometry parameter of the circuit. The plots are created using a set of randomly-generated designs. Only the points for which the corresponding characteristics allow for extracting the approximated operating parameters, as indicated above, are shown. Clear patterns are visible even though the trial points were not optimized whatsoever.

The characteristics points are tailored to address specific design objectives^80^. Take, for example, a microwave coupler like the rat-race structure, and its responses depicted in Fig. 3. To regulate the center frequency, bandwidth of isolation characteristics, and power division ratio, the feature points can be defined as illustrated in Fig. 4, further detailed in the figure caption. Figure 4b demonstrates the straightforward connections between these feature points and the adjustable parameters. For further insights into this topic, additional discussions can be found in references such as^80^ and^86^. In this work, we exploit these properties to facilitate a construction of surrogate models employed to enable and accelerate the global search process.

At this point, it should be emphasized that utilization of response feature adds a layer of complexity in algorithm implementation, which is due to the necessity of defining and extracting the feature points. In practice, this requires implementation of a separate algorithm for scanning the frequency characteristics and identifying the feature points, here, realized in Matlab. On the other hand, especially when the parameter space is very large (broad ranges of geometry parameters) and highly-dimensional, the features might be difficult to identify. The latter normally occurs for heavily distorted responses at poor-quality designs. In practice, designs like these may be assigned high value of the objective function *U*_*F*_ to mark them as low-quality. Nonetheless, the aforementioned issue constitutes a limitation of the proposed approach, yet can be mitigated to a certain extent by appropriate selection of the parameter space (e.g., based on prior experience with a given microwave circuit).

To integrate the response feature approach into the optimization process, we must establish suitable notation. Specifically, we will denote the feature points of the circuit under design as ***f***_*P*_(***x***) = [***f***_*f*_(***x***)^*T*^ ***f***_*L*_(***x***)^*T*^]^*T*^, with ***f***_*f*_(***x***) = [*f*_*f*.1_(***x***) … *f*_*f.K*_(***x***)]^*T*^ and ***f***_*L*_(***x***) = [*f*_*L*.1_(***x***) … *f*_*L.K*_(***x***)]^*T*^ being the vector of frequency and level coordinates, respectively. Table 1 provides a selection of example characteristics points for a microwave coupling circuit, focusing on the device's operating frequency and power split ratio.

**Table 1:** Example response features for a microwave coupler (cf. Fig. 4).

Verbal description	Four feature points: Two points corresponding to the minima of the |*S*_11_| and |*S*_41_| responses Two points corresponding to the power split ratio (i.e., the evaluations of |*S*_31_| and |*S*_21_| at the centre frequency of the circuit)
Vector notation	${\varvec{f}}_{f} ({\varvec{x}}) = \left[ {f_{f.1} ({\varvec{x}})\;\;f_{f.2} ({\varvec{x}})\;\;f_{f.3} ({\varvec{x}})\;\;f_{f.4} ({\varvec{x}})} \right]^{T}$ ${\varvec{f}}_{L} ({\varvec{x}}) = \left[ {f_{L.1} ({\varvec{x}})\;\;f_{L.2} ({\varvec{x}})\;\;f_{L.3} ({\varvec{x}})\;\;f_{L.4} ({\varvec{x}})} \right]^{T}$
Explanation of terms	*f*_*f*.1_ and *f*_*f.*2_—frequencies corresponding to the minima of |*S*_11_| and |*S*_41_| *f*_*L*.1_ and *f*_*L.*2_—corresponding minima levels *f*_*f.*3_ = *f*_*f.*4_ = [*f*_*f*.1_ + *f*_*f.*2_]/2 = *f*_0_—approximated operating frequency of the coupler *f*_*L*.3_ and *f*_*L*.4_—levels of |*S*_31_| and |*S*_41_| at *f*_0_ The power split ratio can be computed as *K*_*P*_ = *f*_*L*.3_ – *f*_*L.*4_

Having ***f***_*P*_, we denote by ***F***_*o*_(***x***) = ***F***_*o*_(***f***_*P*_(***x***)) the circuit’s operating parameters, computed from ***f***_*P*_(***x***). Following this, the design task posed in terms of characteristics points appears as:

2 $$ {\varvec{x}}^{*} = \arg \mathop {\min }\limits_{{\varvec{x}}} U_{F} ({\varvec{x}},{\varvec{f}}_{P} ({\varvec{x}}),{\varvec{F}}_{t} ) $$

The analytical form of the function *U*_*F*_ is similar to that shown in Fig. 2 but employs ***F***_*o*_(***x***) rather than scattering parameters *S*_*kl*_. It can also be written in a compact manner as

3 $$ U_{F} ({\varvec{x}},{\varvec{f}}_{P} ({\varvec{x}}),{\varvec{F}}_{t} ) = U_{0} ({\varvec{f}}_{P} ({\varvec{x}})) + \beta ||{\varvec{F}}_{o} ({\varvec{f}}_{P} ({\varvec{x}})) - {\varvec{F}}_{t} ||^{2} $$

Here, the function *U*_0_ accounts for the main objective. For clarification, consider a scenario, under which we seek to achieve the following: (i) align the coupler center frequency at the target value *f*_*t*_, (ii) establish the target power division ratio, and (iii) reduce |*S*_11_| (impedance matching) and |*S*_41_| (port isolation) at *f*_*t*_. 

In this case, the main goal is to reduce *f*_*L*.1_ and *f*_*L.*2_, cf. Table 1, i.e., we have *U*_0_(***f***_*P*_(***x***)) = max{*f*_*L.*1_(***x***),*f*_*L.*2_(***x***)}. Further, given ***F***_*t*_ = [*f*_*t*_* K*_*P*_]^*T*^, we define ***F***_*o*_(***f***_*P*_(***x***)) = [(*f*_*f*.1_ + *f*_*f*.2_)/2 *f*_*L.*3_ –* f*_*L*.4_]^*T*^. The importance of the operating condition alignment is controlled by the scalar factor β. Typically, we set β = 100. It should be noted that conventional formulation (as shown in Fig. 2) is equivalent to (3) assuming that the optimum is attainable. Further, the second term in (3) (not present in standard formulation) acts as a regularization factor that facilitates identification of the optimum. 

### Variable-fidelity computational models

**Figure 5:**
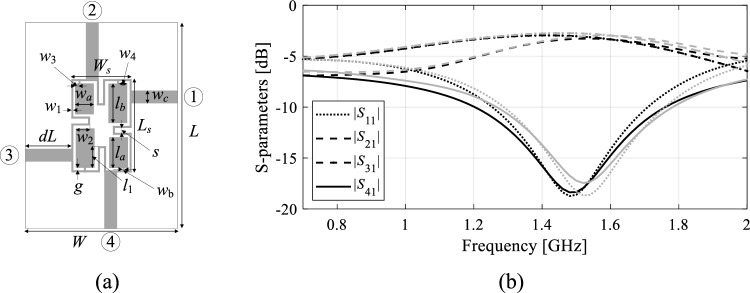
Variable-fidelity models: (**a**) geometry of an exemplary compact branch-line coupler, (**b**) scattering parameters evaluated using the low-fidelity EM model (gray) and the high-fidelity one (black). At the design shown, the simulation time of the high-fidelity model is about 250 s, whereas the evaluation of the low-fidelity model takes about 90 s.

Reducing the EM analysis resolution—by decreasing the structural discretization density (alternative options discussed in^90^)—expedites the simulation process but compromises accuracy, as shown in Fig. 5. This accuracy loss can be rectified by appropriately adjusting the low-fidelity model, forming the basis of physics-based surrogate-assisted methodologies. One prominent example is space mapping^91,92^. The degree of acceleration achievable varies with the problem. For most microwave passive components, it ranges between 2.5 and six for the low-fidelity model, ensuring adequate representation of crucial circuit output details.

In this study, the low- and high-resolution models are denoted as ***R***_*c*_(***x***) and ***R***_*f*_(***x***), respectively. ***R***_*c*_ will be utilized to:

- Pre-screen the parameter space, i.e., to generate a set of random observables, from which those with extractable feature points will be selected for further processing;
- Construct the initial surrogate model using kriging (cf. "Surrogate modelling using kriging and co-kriging" section).

Subsequent optimization steps will employ the low-fidelity samples into a co-kriging surrogate (cf. "Surrogate modelling using kriging and co-kriging" section), which will also incorporate ***R***_*f*_ data acquired during the algorithm run.

At this point, it should be emphasized that the pre-screening process in large parameter spaces (broad ranges of geometry parameters, high dimensionality) may be a limiting factor in terms of the computational efficiency. This is because is large spaces, the percentage of random designs with extractable features might be low, meaning that acquisition of a required number of accepted design would incur considerable expenses. One way of mitigating this issue is appropriate selection of the search space, based on engineering experience and prior knowledge about the circuit at hand, so that reasonably narrow parameter ranges can be established. On the other hand, the aforementioned issue would be also detrimental to any global search algorithm, whether it is a direct method of surrogate-assisted one.

### Surrogate modelling using kriging and co-kriging

Kriging and co-kriging interpolation^93,94^ will be used as the modelling methods of choice within the machine learning framework proposed in this work. Both techniques are briefly recalled below. 

We consider two datasets:

- The low-fidelity one {***x***_*Bc*_^(*k*)^,***R***_*c*_(***x***_*Bc*_^(*k*)^)}_*k* = 1, …, *NBc*_, which consists of the parameter vectors ***x***_*Bc*_^(*k*)^ and the corresponding circuit outputs;
- The high-fidelity set {***x***_*Bf*_^(*k*)^,***R***_*f*_(***x***_*Bf*_^(*k*)^)}_*k* = 1, …, *NBf*_, acquired through evaluation of the high-fidelity model ***R***_*f*_ at parameter vectors ***x***_*Bf*_^(*k*)^.

Tables 2 and 3 outline both kriging and co-kriging models ***s***_*KR*_(***x***) and ***s***_*CO*_(***x***), respectively. Note that the co-kriging surrogate incorporates the kriging model ***s***_*KRc*_ set up based on the low-fidelity data (*X*_*Bc*_, ***R***_*c*_(*X*_*Bc*_)), and ***s***_*KRf*_ generated on the residuals (*X*_*Bf*_, ***r***); here ***r*** = ***R***_*f*_(*X*_*Bf*_) – *ρ*⋅***R***_*c*_(*X*_*Bf*_). The parameter *ρ* is included in the Maximum Likelihood Estimation (MLE) of the second model^94^. If the low-fidelity data at *X*_*Bf*_ is unavailable, one can use an approximation ***R***_*c*_(*X*_*Bf*_) ≈ ***s***_*KRc*_(*X*_*Bf*_). The same correlation function is utilized by both models (cf. Tables 2 and 3).

**Table 2:** An outline of the kriging surrogate modeling. More information can be found in the literature, e.g.,^94^.

Model component	Analytical form
Model formulation	${\varvec{s}}_{KR} ({\varvec{x}}) = {\varvec{M}}\gamma + r({\varvec{x}}) \cdot {{\varvec{\Psi}}}^{ - 1} \cdot ({\varvec{R}}_{c} (X_{Bc} ) - {\varvec{F}}\gamma )$ where ***M*** is a *N*_*Bc*_ × *t* model matrix of *X*_*Bc*_, whereas ***F*** is a 1 × *t* vector of the evaluation point ***x*** (*t* is the number of terms used in the regression function^91^)
Regression function coefficients	$\gamma = (X_{Bc}^{T} {{\varvec{\Psi}}}^{ - 1} X_{Bc} )^{ - 1} X_{Bc} {{\varvec{\Psi}}}^{ - 1} {\varvec{R}}_{f} (X_{Bc} )$
1 × *N*_*Bc*_ vector of correlations between ***x*** and *X*_*Bc*_	$r({\varvec{x}}) = (\psi ({\varvec{x}},{\varvec{x}}_{Bc}^{(1)} ),...,\psi ({\varvec{x}},{\varvec{x}}_{Bc}^{{(N_{Bc} )}} ))$
Correlation matrix	***Ψ*** = [Ψ_*i*,*j*_] is a correlation matrix, where Ψ_*i*,*j*_ = *ψ*(***x***_*Bf*_^(*i*)^,***x***_*Bf*_^(*j*)^)
Correlation function	$\psi ({\varvec{x}},{\varvec{x}}^{\prime}) = \exp \left( {\sum\nolimits_{k = 1}^{n} { - \theta_{k} |x^{k} - x^{^{\prime}k} |^{P} } } \right)$
Model identification: finding hyperparameters *θ*_*k*_, *k* = 1, …, *n*, using Maximum Likelihood Estimation (MLE^94^)	$(\theta_{1} ,...,\theta_{n} ) = \arg \mathop {\min }\limits_{{\theta_{1} ,...,\theta_{n} }} \left[ { - (N_{Bf} /2)\ln (\hat{\sigma }^{2} ) - 0.5\ln (|\Psi |)} \right]$ where $\hat{\sigma }^{2} = ({\varvec{R}}_{f} (X_{Bf} ) - F\alpha )^{T} {{\varvec{\Psi}}}^{ - 1} ({\varvec{R}}_{f} (X_{Bf} ) - F\alpha )/N_{Bf}$ and |***Ψ***| is the determinant of ***Ψ***. In practice, a Gaussian correlation function (*P* = 2) is often employed, as well as ***F*** = [1 … 1]^*T*^ and ***M*** = 1

**Table 3:** An outline of the co-kriging surrogate modeling.

Model component	Analytical form
Model formulation	${\varvec{s}}_{CO} ({\varvec{x}}) = {\varvec{M}}\gamma + r({\varvec{x}}) \cdot {{\varvec{\Psi}}}^{ - 1} \cdot ({\varvec{r}} - {\varvec{F}}\gamma )$
Vector of correlations	$r({\varvec{x}}) = [\rho \cdot \sigma_{c}^{2} \cdot r_{c} ({\varvec{x}}),\rho^{2} \cdot \sigma_{c}^{2} \cdot r_{c} ({\varvec{x}},X_{{B_{f} }} ) + \sigma_{d}^{2} \cdot r_{d} ({\varvec{x}})]$
Correlation matrix	$${{\varvec{\Psi}}} = \left[ {\begin{array}{*{20}c} {\sigma_{c}^{2} {{\varvec{\Psi}}}_{c} (X_{Bc} ,X_{Bc} )} & {\rho \,\sigma_{c}^{2} {{\varvec{\Psi}}}_{c} (X_{Bc} ,X_{Bf} )\,} \\ {\rho \,\sigma_{c}^{2} {{\varvec{\Psi}}}_{c} (X_{Bf} ,X_{Bc} )} & {\rho^{2} \sigma_{c}^{2} {{\varvec{\Psi}}}_{c} (X_{Bf} ,X_{Bf} ) + \sigma_{d}^{2} {{\varvec{\Psi}}}_{d} } \\ \end{array} } \right]$$ where and ***M*** = [*ρ****M***_*c*_ ***M***_*d*_] where (***F***_*c*_, *σ*_*c*_, ***Ψ***_*c*_, ***M***_*c*_) and (***F***_*d*_, *σ*_*d*_, ***Ψ***_*d*_, ***M***_*d*_) are matrices obtained from ***s***_*KRc*_ and ***s***_*KRf*_, respectively^91^; parameter *ρ* is included in the MLE during model identification

More information can be found in the literature, e.g.,^94^.

### Parameter space pre-screening. Initial surrogate model construction

The initial stage of the machine learning process described in this paper involves screening the parameter space, conducted at the level of low-fidelity EM models. Subsequently, an initial surrogate model is developed, utilizing the low-fidelity data collected during this phase. The pre-screening stage operates at the response feature level (refer to "Response feature methodology" section), enabling a sizeable curtailment of the number of samples compared to analysing the complete frequency characteristics of the designed circuit.

The primary aim of the initial surrogate ***s***^(0)^(***x***) is to capture the behaviour exhibited by the frequency and level coordinates of the feature points delineated for the optimized circuit. We have

4 $$ {\varvec{s}}^{(0)} \left( {\varvec{x}} \right) = \left[ {\left[ {s_{f.1}^{\left( 0 \right)} \left( {\varvec{x}} \right) \ldots s_{f.K}^{\left( 0 \right)} \left( {\varvec{x}} \right)} \right]^{T} \left[ {s_{L.1}^{\left( 0 \right)} \left( {\varvec{x}} \right) \ldots s_{L.K}^{\left( 0 \right)} \left( {\varvec{x}} \right)} \right]^{T} } \right]^{T} $$

The model is rendered by means of kriging^95^ (cf. "Surrogate modelling using kriging and co-kriging" section, Table 2). The training data pairs are denoted as {***x***_*Bc*_^(*j*)^,***f***_*P*_(***x***_*B*_^(*j*)^)}, *j* = 1, …, *N*_*init*_. The points are generated randomly in the parameter space *X* using independent joint uniform probability distributions; only the observables with extractable response features are included into the training set. The dataset size *N*_*init*_ is adjusted to secure a sufficient surrogate model accuracy. For quantification, we utilize the relative RMS error, wherein the acceptance threshold *E*_max_ serves as a controlling parameter within the process. Given the characteristics of the response feature, the demand for training points to establish a dependable model remains minimal, typically ranging from fifty to one hundred. Depending on the parameter space's dimensionality and ranges, the ratio of accepted observables falls between twenty to seventy percent. Consequently, the actual count of low-fidelity EM simulations required to compile the dataset {***x***_*Bc*_^(*j*)^,***f***_*P*_(***x***_*B*_^(*j*)^)} varies from 1.5*N*_*init*_ to 5*N*_*init*_. In practice, the process of rejecting (possibly significant) subset of the random samples acts as a pre-selection mechanism: it enables the identification of promising regions within the parameter space, concentrating the search process on these areas while excluding others. The pre-screening procedure has been summarized in Table 4.

**Table 4:**
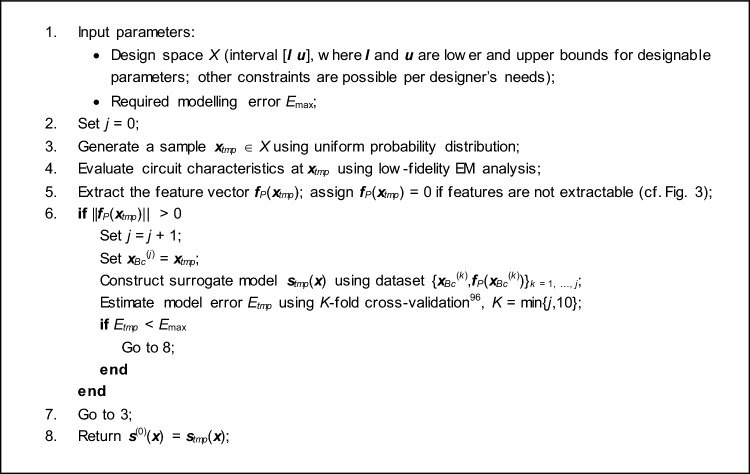
Parameter space pre-screening and initial surrogate model construction. A pseudocode.

### Generating infill by means of nature-inspired optimization. Co-kriging surrogate

The core part of the optimization run consists of iterative generation of candidate solutions ***x***^(*i*)^, *i* = 1, 2, …, to the problem (2), (3), and construction of the refined surrogate models ***s***^(*j*)^, *j* = 1, 2, … . This stage is carried out using the high-resolution model ***R***_*f*_. Each iteration produces a new design

5 $$ {\varvec{x}}^{(i + 1)} = \arg \mathop {\min }\limits_{{{\varvec{x}} \in X}} U_{F} ({\varvec{x}},{\varvec{s}}^{(i)} ({\varvec{x}}),{\varvec{F}}_{t} ) $$

using the current surrogate ***s***^(*i*)^, which is a co-kriging model (cf. "Parameter space pre-screening. Initial surrogate model construction" section) constructed using the low-fidelity dataset {***x***_*Bc*_^(*j*)^, ***f***_*P*_(***x***_*Bc*_^(*j*)^)}, *j* = 1, …, *N*_*init*_, and the high-fidelity dataset {***x***_*f*_^(*j*)^, ***f***_*P*_(***x***_*f*_^(*j*)^)}, *j* = 1, …, *i*, consisting of the samples accumulated until iteration *i*.

The formulation of the sub-problem (5) is the same as for the task (2), except that the response features are obtained from ***s***^(*i*)^. Optimization is conducted in a global sense using the particle swarm optimization (PSO) algorithm^97^. PSO was chosen as a representative nature-inspired method, which also belongs to the most popular ones. Yet, any other population-based technique can be used instead because operating at the level of fast surrogate does not imposes any practical constraints on the computational budget expressed as the number of objective function calls. Furthermore, finding the global optimum of *U*_*F*_(***x***,***s***^(*i*)^(***x***),***F***_*t*_) is considerably easier handling the original merit function *U*(***x***,***F***_*t*_) due to inherent regularization that comes with the employment of response features.

In view of machine learning, generating candidate designs using (5) corresponds to the infill criterion being the predicted objective function improvement^98^. Upon completing the pre-screening stage, the parameter space subset containing the optimum has been presumably identified, therefore, the search process can now be focused on its exploitation, rather than enhancing the overall metamodel’s accuracy. The algorithm is stopped either due to convergence in argument, i.e., ||***x***^(*i*+1)^ – ***x***^(*i*)^||< *ε* or if no improvement of the objective function has been detected over the last *N*_*no_improve*_ iterations, whichever occurs first. The default values of the termination thresholds are *ε* = 10^–2^ and *N*_*no_improve*_ = 10.

### Complete optimization framework

**Figure 6:**
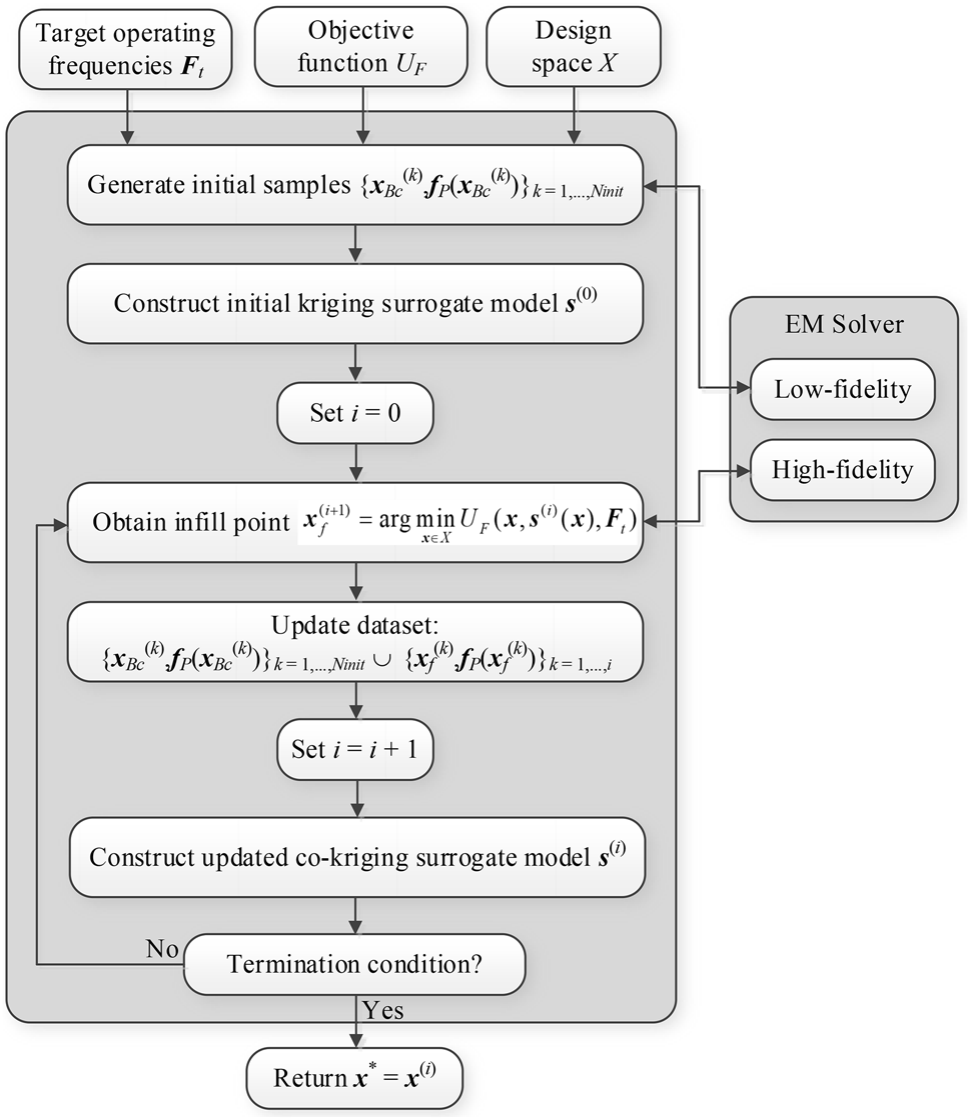
Flow diagram of the proposed ML algorithm for global optimization of microwave passive components.

The proposed global optimization framework is outlined in Table 5 as a pseudocode and depicted in Fig. 6 as a flow diagram. The pre-screening stage unfolds within Steps 2 and 3, while the heart of the search process—creating infill points and refining the surrogate model—is executed across Steps 4 through 8. Step 9 verifies the termination criteria.

**Table 5:**
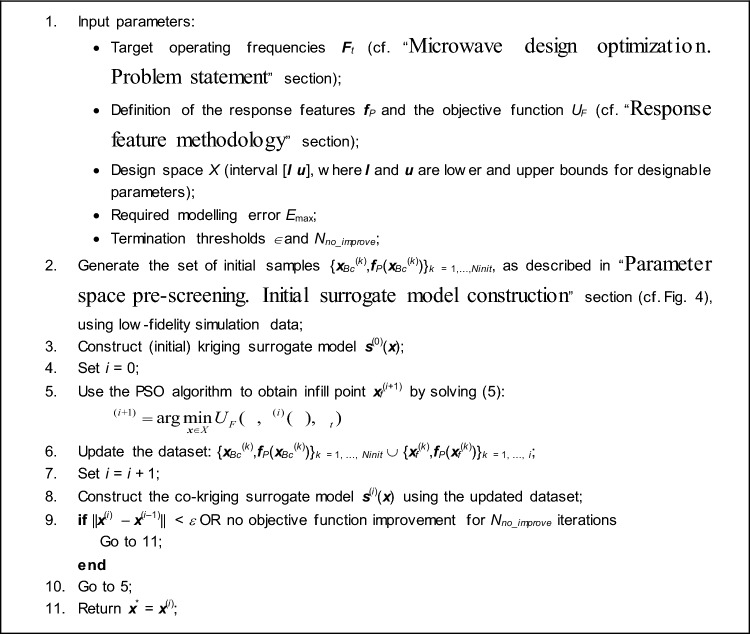
Pseudocode of the ML framework for feature-based global parameter tuning of microwave components introduced in this study.

Let us discuss the control parameters compiled in Table 6. It is essential to note that there are only three parameters, with two determining the termination conditions. Their primary function is to govern the resolution of the optimization process. The third parameter manages the accuracy of the initial surrogate. The default value of *E*_max_ corresponds to ten percent of the relative RMS error, which is a mild condition. A small number of control parameters is an advantage of the method as it eliminates the need for tailoring the setup to the particular being solved. In fact, identical arrangement will be used for all verification experiments described in "Verification experiments and benchmarking" section.

**Table 6:** Control parameters of the proposed ML algorithm for global optimization microwave passives.

Parameter	Meaning	Default value
*E*_max_	Maximum value of relative RMS error of the initial surrogate model (error estimated using cross-validation), cf. "Parameter space pre-screening. Initial surrogate model construction" section	10%
*ε*	Termination threshold for convergence in argument, cf. "Generating infill by means of nature-inspired optimization. Co-kriging surrogate" section	10^–2^
*N*_*no_improve*_	Termination threshold for no objective function value improvement, cf. "Generating infill by means of nature-inspired optimization. Co-kriging surrogate" section	10

The full-wave electromagnetic simulations were performed on Intel Xeon 2.1 GHz dual-core CPU with 128 GB RAM, using CST Microwave Studio. The optimization framework has been implemented in MATLAB. The particle swarm optimizer and CST simulation software communicate through a Matlab-CST socket. The kriging and co-kriging surrogate models were set up using the SUMO toolbox of^99^. As mentioned earlier, the underlying optimization engine is particle swarm optimizer PSO^100^, which is one of the most representative nature-inspired population-based algorithms. PSO processes a swarm of *N* particles (parameter vectors) ***x***_*i*_ and velocity vectors ***v***_*i*_, which stand for the position and the velocity of the *i*th particle, respectively. These are updated as follows:

6 $$ {\varvec{v}}_{i} \leftarrow \chi [{\varvec{v}}_{i} + c_{1} {\varvec{r}}_{1} \cdot ({\varvec{x}}_{i}^{*} - {\varvec{x}}_{i} ) + c_{2} {\varvec{r}}_{2} \cdot ({\varvec{g}} - {\varvec{x}}_{i} )] $$

7 $$ {\varvec{x}}_{i} \leftarrow {\varvec{x}}_{i} + {\varvec{v}}_{i} $$

where ***r***_1_ and ***r***_2_ are vectors whose components are uniformly distributed random numbers between 0 and 1; · denotes component-wise multiplication. In our numerical experiments we use a standard setup:

- Size of the swarm *N* = 10,
- Maximum number of iterations *k*_max_ = 100,
- Control parameters, *χ* = 0.73, *c*_1_ = *c*_2_ = 2.05, cf.^100^.

As indicated in (7), the first step of altering the positions ***x***_*i*_ of the particles is the adjustment of the velocity vector, which is partially stochastic. There are three components therein, one being the current velocity, the second fostering particle relocation towards its local best position ***x***_i_^*^, and the third one pushing the particle towards global best position ***g*** found so far by the swarm. The mentioned setup of control parameters is the most widely used one, typically recommended in the literature^99^.

## Verification experiments and benchmarking

The validation of the optimization algorithm outlined in "Global machine-learning microwave optimization using response features and multi-resolution computational models" section involves numerical testing with two microstrip circuits. For comparison, these devices are also optimized with the use of several benchmark methods that include a gradient-based search with random starting point, a particle swarm optimizer (PSO), but also two machine-learning frameworks: (i) a procedure that directly handles frequency characteristics of the circuit, and (ii) the algorithm of "Global machine-learning microwave optimization using response features and multi-resolution computational models" section exclusively using high-fidelity EM simulations. We chose these specific methods to showcase the multimodal nature of the design challenges and to validate the significance of the algorithmic tools embedded in the proposed procedure—particularly the use of response features and variable-fidelity simulations. The performance evaluation criteria encompass the optimization process's reliability (quantified as a success rate, i.e., the proportion of algorithm runs yielding acceptable outcomes), design quality, and computational efficiency.

The material in this section is arranged as follows: "Verification circuits" section offers insights into the verification structures. The experimental setup and results are outlined in "Experimental setup. Numerical results" section, while "Discussion" section delves into the characteristics of the techniques considered and encapsulates the overall performance of the proposed approach.

### Verification circuits

The machine learning procedure introduced in this study is demonstrated using two planar structures:

- A miniaturized rat-race coupler (RRC) with meandered transmission lines (Circuit I)^101^;
- A dual-band power divider with equal division ratio (Circuit II)^102^.

**Figure 7:**
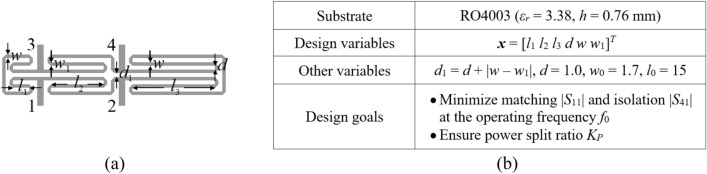
Circuit I (rat-race coupler with folded transmission lines)^101^: (**a**) geometry, (**b**) essential parameters.

**Figure 8:**
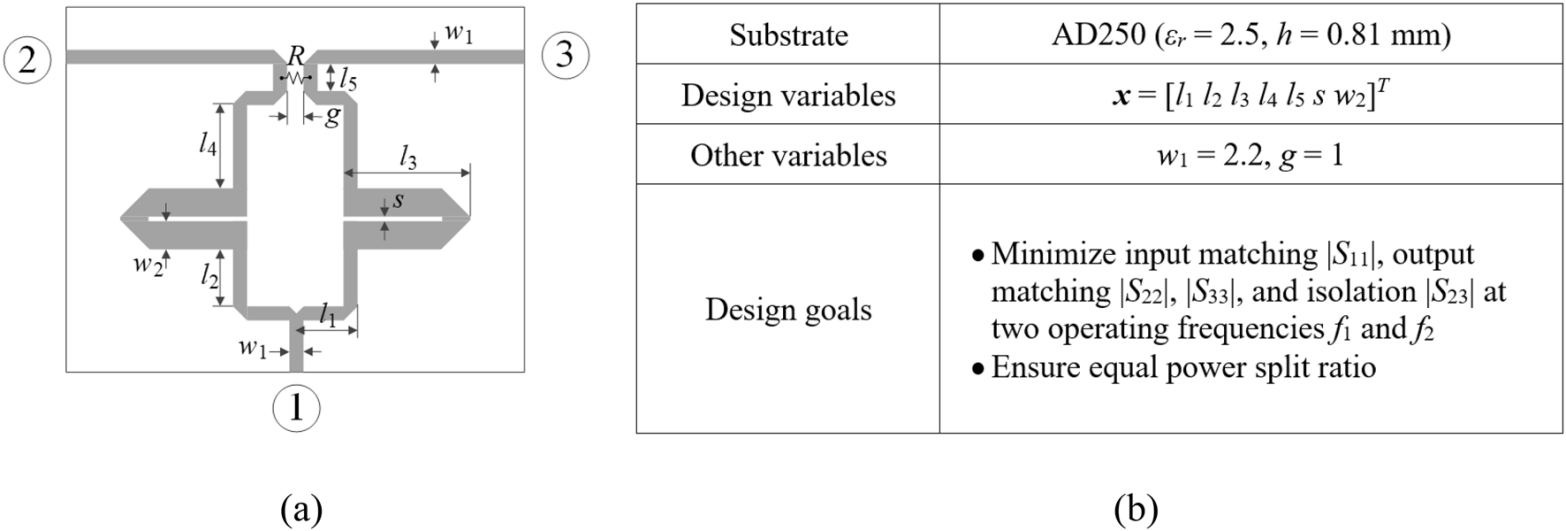
Circuit II (dual-band equal-split power divider)^102^: (**a**) geometry, (**b**) essential parameters.

The circuit topologies are depicted in Figs. 7a and 8a, respectively. Essential data for both circuits, including substrate parameters, design variables, and design goals, are detailed in Figs. 7b and 8b. The computational models are simulated in CST Microwave Studio, employing the time-domain solver. The low-fidelity models are representations with coarser discretization compared to the high-fidelity versions. For specific details regarding the number of mesh cells and simulation times for ***R***_*c*_ and ***R***_*f*_, please refer to Table 7.

**Table 7:** EM simulation models for Circuit I and II.

Circuit	EM simulation model			
	Low-fidelity ***R***_*c*_		High-fidelity ***R***_*f*_	
	Discretization density (# of mesh cells)	Simulation time [s]	Discretization density (# of mesh cells)	Simulation time [s]
I	~ 50,000	50	~ 180,000	120
II	~ 130,000	60	~ 570,000	310

The design goals for have been set as follows:

- *Circuit I* (i) enhance the matching and isolation responses, |*S*_11_| and |*S*_41_|, at the required frequency *f*_0_; (ii) realize required power division |*S*_31_| – |*S*_21_|= *K*_*P*_ [dB] (also at *f*_0_).
- *Circuit II* (i) enhance input impedance matching |*S*_11_|, output matching |*S*_22_|, |*S*_33_|, and isolation |*S*_23_| at the frequencies *f*_1_ and *f*_2_ corresponding to the two operating bands of the circuit, (ii) ensure equal power division at *f*_1_ and *f*_2_. Here, the second condition automatically follows from the circuit symmetry.

In order to expand the scope of the verification experiments, two design scenarios are considered for each circuit as detailed in Table 8. One can also notice that the search spaces, determined by the bounds on design variables, are extensive: the (mean) upper-to-lower bound ratio is 13 and 5 for Circuit I and II, respectively.

**Table 8:** Design goals and parameter spaces for Circuits I and II.

Circuit	Target operating parameters		Parameter space *X* (lower bounds ***l*** and upper bounds ***u***)
	Symbols	Specific values for numerical experiments	
I	***F***_*t*_ = [*f*_0_ *K*_*P*_]^*T*^	Case 1: *f*_0_ = 1.8 GHz, *K*_*P*_ = − 3 dB	***l*** = [0.5 5.0 5.0 0.2 0.2 0.2]^*T*^ ***u*** = [15.0 30.0 50.0 2.0 2.0 2.0]^*T*^
		Case 2: *f*_0_ = 1.2 GHz, *K*_*P*_ = 0 dB	
II	***F***_*t*_ = [*f*_1_ *f*_2_]^*T*^	Case 1: *f*_1_ = 3.0 GHz, *f*_2_ = 4.8 GHz	***l*** = [10.0 1.0 10.0 0.5 1.0 0.1 1.5]^*T*^ ***u*** = [40.0 20.0 40.0 15.0 6.0 1.5 8.0]^*T*^
		Case 2: *f*_1_ = 2.0 GHz, *f*_2_ = 3.3 GHz	

### Experimental setup. Numerical results

Circuits I and II were optimized by means of the procedure proposed in this study. The setup uses default values of control parameters, as specified in Table 6: *E*_max_ = 10%, *ε* = 10^–2^, and *N*_*no_improve*_ = 10. It is kept identical for all considered design cases. For the purpose of comparison, our verification structures were also optimized using several benchmark methods. The technical details on these techniques can be found in Table 9. Below, we explain the reasons for selecting this particular testbed:

- Algorithm I: particle swarm optimizer (PSO), chosen as one of the most popular nature-inspired algorithms to date. Its inclusion in the benchmark set aims to showcase the difficulties associated with direct EM-driven population-based optimization. For practical reasons, PSO is run at low computational budgets of 500 (version I) and 1000 objective function evaluations (version II), yet, the computational costs entailed even with these budgets are at the edge of being prohibitive;
- Algorithm II: A gradient-based search with random initial design. This method is selected to illustrate multi-modality of our optimization tasks, thereby indicating the need for employing global search methodologies;
- Algorithm III: Machine-learning procedure processing complete frequency responses of the circuit at hand. The algorithm utilizes the similar components as those described in "Global machine-learning microwave optimization using response features and multi-resolution computational models" section (kriging surrogates, predicted objective function improvement) but works exclusively with the high-resolution EM model. This method is selected to investigate the benefits of incorporating response features.
- Algorithm IV: Feature-based machine-learning procedure similar to that described in "Global machine-learning microwave optimization using response features and multi-resolution computational models" section, but only using high-fidelity EM models. Consequently, kriging surrogate is employed throughout the entire optimization run instead of co-kriging. This approach serves to highlight the benefits of integrating variable-fidelity models into the method presented in this work.

**Table 9:** Benchmark algorithms: the outline.

Algorithm	Algorithm type	Setup
I	Particle swarm optimizer (PSO)	Swarm size *N* = 10, standard control parameters (*χ* = 0.73, *c*_1_ = *c*_2_ = 2.05); number of iterations set to 50 (version I) and 100 (version II)
II	Trust-region gradient based optimizer^103^	Random initial design, response gradients estimated using finite differentiation, termination criteria based on convergence in argument and reduction of the trust region size^103^
III	Machine learning algorithm (cf. "Global machine-learning microwave optimization using response features and multi-resolution computational models" section)	Algorithm similar to that of "Global machine-learning microwave optimization using response features and multi-resolution computational models" section Initial surrogate set up to ensure relative RMS error not higher than 10% with the maximum number of training samples equal to 400; Optimization based on processing the antenna frequency characteristics (unlike response features in the proposed procedure); Infill criterion: minimization of the projected objective function improvement^98^
IV	Feature-based machine learning algorithm utilizing high-fidelity EM simulations only	Algorithm highlights Surrogate model constructed at the level of response features; Optimization process only uses high-fidelity EM simulations; Infill criterion: minimization of the predicted objective function^98^

**Figure 9:**
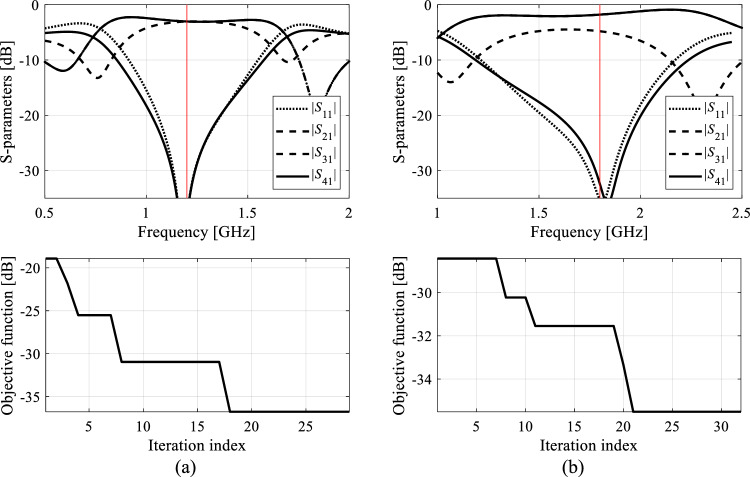
Exemplary runs of the proposed machine-learning framework. Shown are: *S*-parameters of Circuit I (Case 1) at the designs produced by the proposed technique (top), and the evolution of the objective function value (bottom): (**a**) run 1, (**b**) run 2. The iteration counter starts after constructing the initial surrogate model. Target operating frequency, here, 1.8 GHz, marked using the vertical lines.

**Figure 10:**
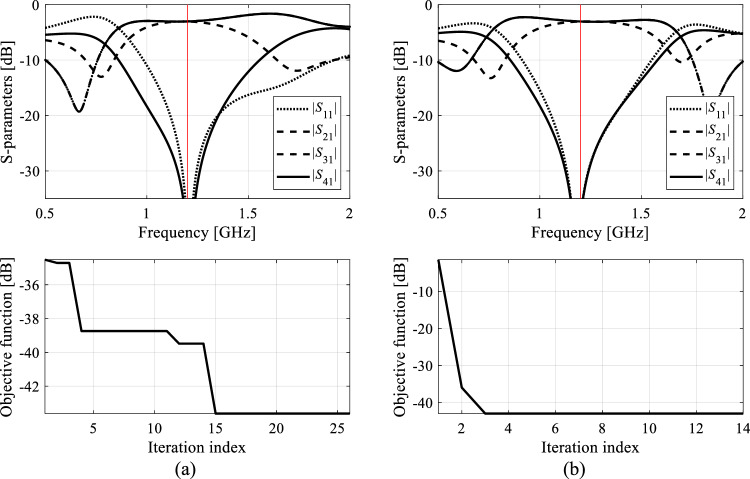
Exemplary runs of the proposed machine-learning framework. Shown are: *S*-parameters of Circuit I (Case 2) at the designs produced by the proposed technique (top), and the evolution of the objective function value (bottom): (**a**) run 1, (**b**) run 2. The iteration counter starts after constructing the initial surrogate model. Target operating frequency, here, 1.2 GHz, marked using vertical lines.

**Figure 11:**
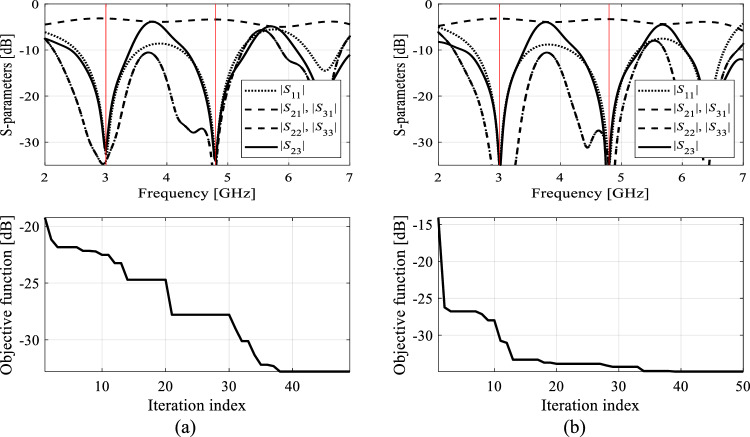
Exemplary runs of the proposed machine-learning framework. Shown are: *S*-parameters of Circuit II (Case 1) at the designs produced by the proposed technique (top), and the evolution of the objective function value (bottom): (**a**) run 1, (**b**) run 2. The iteration counter starts after constructing the initial surrogate model. Target operating frequencies, 3.0 GHz and 4.8 GHz, marked using vertical lines.

**Figure 12:**
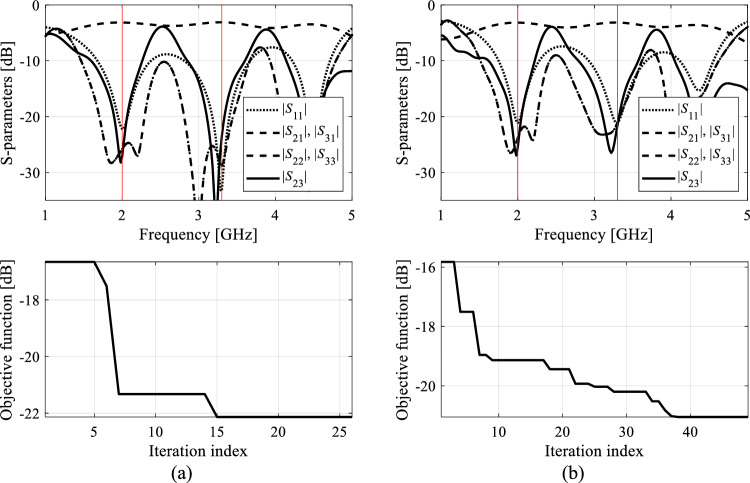
Exemplary runs of the proposed machine-learning framework. Shown are: *S*-parameters of Circuit II (Case 2) at the designs produced by the proposed technique (top), and the evolution of the objective function value (bottom): (**a**) run 1, (**b**) run 2. The iteration counter starts after constructing the initial surrogate model. Target operating frequencies, 2.0 GHz and 3.3 GHz, marked using vertical lines.

Each algorithm underwent ten executions, and the average outcomes (merit function value, computational expenses) are documented in Tables 10 and 11 for Circuit I and II, respectively. The success rate denotes the number of runs (out of ten) generating designs that sufficiently match their target operating parameters. Figures 9, 10, 11, and 12 exhibit the circuit responses and the progression of the objective function value across both design cases and chosen algorithm runs.

**Table 10:** Optimization of Circuit I: results.

Optimization algorithm		Performance figure		
		Average objective function value [dB]	Computational cost^a^	Success rate^b^
Case 1 *f*_0_ = 1.8 GHz, *K*_*P*_ = − 3 dB	Algorithm I: PSO (50 iterations)	− 24.8	500	9/10
	Algorithm I: PSO (100 iterations)	− 34.0	1000	10/10
	Algorithm II: Trust-region gradient-based algorithm	− 18.7	102.8	6/10
	Algorithm III: Machine learning procedure handling complete circuit responses	− 34.1	435.7	10/10
	Algorithm IV: Feature-based machine learning procedure using high-fidelity model only	− 34.8	89.9	10/10
	Proposed algorithm (feature-based machine learning with variable-fidelity simulation models)	− 34.5	48.2	10/10
Case 2 *f*_0_ = 1.2 GHz, *K*_*P*_ = 0 dB	Algorithm I: PSO (50 iterations)	− 23.7	500	9/10
	Algorithm I: PSO (100 iterations)	− 36.2	1000	10/10
	Algorithm II: Trust-region gradient-based algorithm	48.3	68.7	5/10
	Algorithm III: Machine learning procedure handling complete circuit responses	− 45.6	433.6	10/10
	Algorithm IV: Feature-based machine learning procedure using high-fidelity model only	− 43.9	106.3	10/10
	Proposed algorithm (feature-based machine learning with variable-fidelity simulation models)	− 44.1	59.3	10/10

^a^The cost expressed in terms of the number of high-fidelity EM simulations of the circuit under design.

^b^Number of algorithms runs at which the operating frequency was allocated near the target value.

**Table 11:** Optimization of Circuit II: results.

Optimization algorithm		Performance figure		
		Average objective function value [dB]	Computational cost^a^	Success rate^b^
Case 1 *f*_1_ = 3.0 GHz, *f*_2_ = 4.8 GHz	Algorithm I: PSO (50 iterations)	− 19.6	500	8/10
	Algorithm I: PSO (100 iterations)	− 18.8	1000	9/10
	Algorithm II: Trust-region gradient-based algorithm	− 12.3	95.1	2/10
	Algorithm III: Machine learning procedure handling complete circuit responses	− 32.0	446.8	10/10
	Algorithm IV: Feature-based machine learning procedure using high-fidelity model only	− 32.8	194.4	10/10
	Proposed algorithm (feature-based machine learning with variable-fidelity simulation models)	− 32.3	85.2	10/10
Case 2 *f*_1_ = 2.0 GHz, *f*_2_ = 3.3 GHz	Algorithm I: PSO (50 iterations)	− 18.8	500	8/10
	Algorithm I: PSO (100 iterations)	− 19.7	1000	9/10
	Algorithm II: Trust-region gradient-based algorithm	− 20.6	93.8	7/10
	Algorithm III: Machine learning procedure handling complete circuit responses	− 22.5	430.5	10/10
	Algorithm IV: Feature-based machine learning procedure using high-fidelity model only	− 21.1	153.2	10/10
	Proposed algorithm (feature-based machine learning with variable-fidelity simulation models)	− 21.9	52.4	10/10

^a^The cost expressed in terms of the number of high-fidelity EM simulations of the circuit under design.

^b^Number of algorithms runs at which the operating frequency was allocated near the target value.

### Discussion

The data presented in Tables 10 and 11 are examined here to evaluate the efficiency of the suggested machine-learning approach and to juxtapose it with benchmark techniques. We're particularly focused on evaluating the following metrics: the search process's reliability assessed through the success rate, the design quality evaluated using the merit function value, and the cost efficiency of the global search process.

Another point for discussion is the effect of incorporating the response features and variable-fidelity simulation models. The observations are as follows:

- *Search process reliability* Reliability is measured using the success rate (right-hand-side columns of Tables 10 and 11), which is the number of algorithm runs yielding the designs that feature operating parameters being close to the targets. For the proposed method, the success rate is 10/10, just as it is for both benchmark machine learning methods (Algorithms III and IV) and PSO (Algorithm I) set up with the budget of 1000 objective function calls. However, PSO working with the computational budget of 500 function evaluations does not perform as well. Also, for Circuit II, which is a more challenging of the two test problems, the success rate of PSO is 9/10 even the budget of 1000 function calls. This indicates that direct nature-inspired optimization needs higher budgets (e.g., > 2000) to ensure the perfect score. At the same time, gradient-based optimization routinely fails (e.g., the success rate is only 2/10 for Circuit II, Case 1), which corroborates multimodality of the considered design tasks.
- *Design quality* The solutions produced using the presented procedure exhibit comparable quality to those produced by other benchmark methods, as indicated by the average objective function value with the exception of gradient-based search, where the failed algorithm runs degrade the average. In terms of absolute numbers, all machine learning methods deliver results that are sufficiently good for practical applications, i.e., matching and isolation below − 20 dB, and power division close to the required values of 0 dB and 3 dB for Circuits I and II, respectively
- *Computational efficiency* The efficiency of the proposed framework is by far the best across the entire benchmark set and the considered test problems. We omit comparison with gradient-based algorithms as they were included in the benchmark solely to highlight the necessity of global optimization for the test problems considered. The average computational cost, quantified in terms of equivalent high-fidelity EM simulations, is approximately sixty. This signifies considerable savings compared to PSO (with the budget of 1000 function calls) are between 90 and 95 percent, depending on the test case. The savings over Algorithm II (machine learning processing full circuit responses) are about 85 percent on the average, and about 50 percent over Algorithm III (feature-based machine learning working at single-fidelity EM level). Regarding the latter, the average savings are 45 and over sixty percent for Circuit I and II, respectively, because the time evaluation ratio between the high- and low-fidelity model is more advantageous for the latter (5.2 versus 2.4).
- The above numbers confirm that the employment of both response features and variable-fidelity are instrumental in expediting the search process without being detrimental to its reliability. The speedup obtained due to variable-fidelity modeling is significant also because the majority of the circuit evaluations are associated with the parameter space pre-screening, which, in the proposed methods, is conducted using the low-fidelity system representation. A better perspective of the computational benefits due to the mentioned mechanisms can be provided by considering the acceleration factors. For example, the proposed methods is over 17 times faster than PSO, almost eight times faster than Algorithm III, and over twice as fast as Algorithm IV.

The overall performance of the presented methodology is quite promising. The variable-fidelity feature-based machine learning yields consistent results at remarkably low computational cost. Consequently, it seems to be suitable for replacing less efficient global optimization methods in the field of high-frequency EM-driven design. At this point, we should also discuss its potential limitation, which is related to the response feature aspect of the procedure. On one hand, defining and extracting characteristic points from the system outputs for a specific structure and design task adds complexity to the implementation process, albeit not in its core segment, which remains problem independent.

On the other hand, for highly-dimensional problems and very broad parameter ranges, the number of random observables generated in the pre-screening stage may be large as compared to those that exhibit extractable features. Such factors would compromise the computational efficiency of the methods, impacting their overall performance. Naturally, these same factors would also impede the efficacy of all benchmark methods. Yet, assuming that the parameter space is defined by an experienced designer, i.e., it is not excessively large, the risk of the occurrence of the aforementioned issue is rather low.

## Conclusion

This paper introduced a machine-learning framework designed for the efficient global optimization of passive microwave components. Our methodology integrates several crucial mechanisms pivotal for achieving competitive reliability and minimizing search process expenses. These mechanisms encompass the response feature approach, parameter space pre-screening, and the utilization of variable-fidelity EM simulations. The response feature method helps in regularizing the objective function landscape, consequently reducing the necessary dataset size for constructing precise surrogate models. Pre-screening of the parameter space aids in the initial identification of the most promising regions, while variable-resolution models contribute to additional computational acceleration. Within this framework, both low- and high-fidelity simulation data are combined into a unified surrogate model using co-kriging. The optimization process itself focuses on rapid identification of the optimum design, which is facilitated by the infill criterion applied in our framework (predicted objective function improvement). The particle swarm optimization (PSO) algorithm serves as the underlying search engine. Numerical verification experiments involving two microstrip components illustrate the superior performance of the proposed technique, showcasing its ability to achieve superior design quality, reliability, and computational efficiency. The CPU savings versus nature-inspired optimization are up to 95 percent (average acceleration factor of 17), 85 percent over the machine learning procedure working directly with circuit frequency characteristics (acceleration factor of eight), and 50 percent over the feature-based machine learning algorithm that only uses high-fidelity EM models (acceleration factor of two). The mean running cost corresponds to sixty high-resolution EM simulations. This level of expenses is comparable to local optimization. Consequently, the presented framework seems to be an attractive alternative to both conventional and surrogate-assisted global optimization procedures (including machine-learning algorithms) utilized so far in high-frequency engineering. A primary objective for future work involves extending the method to encompass other types of microwave components, such as filters. This extension would involve automating the procedures for defining and extracting feature points. Furthermore, utilization of alternative machine learning technique will be considered, oriented towards improving the reliability of surrogate model construction as well as computational efficiency of the modelling process. Some of the tools to be employed include deep learning methods (e.g., convolutional neural networks, etc.). Finally, the properties of the presented technique will be investigated when applied to higher-dimensional problems (ten design variables and more), and enhancements wil be developed to facilitate operation of the framework under such challenging scenarios.

## Data Availability

The datasets used and/or analysed during the current study available from the corresponding author on reasonable request.
